# Role of Fibroblast Growth Factors in the Crosstalk of Hepatic Stellate Cells and Uveal Melanoma Cells in the Liver Metastatic Niche

**DOI:** 10.3390/ijms231911524

**Published:** 2022-09-29

**Authors:** Tatjana Seitz, Nora John, Judith Sommer, Peter Dietrich, Wolfgang E. Thasler, Arndt Hartmann, Katja Evert, Sven A. Lang, Anja Bosserhoff, Claus Hellerbrand

**Affiliations:** 1 Institute of Biochemistry, Friedrich-Alexander-Universität Erlangen-Nürnberg, D-91054 Erlangen, Germany; 2 Department of Medicine 1, University Hospital Erlangen, Friedrich-Alexander-Universität Erlangen-Nürnberg, D-91054 Erlangen, Germany; 3 Hepacult GmbH, Martinsried, D-82152 Planegg, Germany; 4 Institute of Pathology, University Hospital Erlangen, Friedrich-Alexander-Universität Erlangen-Nürnberg, D-91054 Erlangen, Germany; 5 Comprehensive Cancer Center (CCC) Erlangen-EMN, D-91054 Erlangen, Germany; 6 Institute of Pathology, University of Regensburg, D-93053 Regensburg, Germany; 7 Department of General, Visceral and Transplantation Surgery, University Hospital Rheinisch-Westfälisch Technische Hochschule Aachen, D-52074 Aachen, Germany

**Keywords:** fibroblast growth factors, fibroblast growth factor 9, uveal melanoma, hepatic metastasis, hepatic stellate cells

## Abstract

Hepatic metastasis is the critical factor determining tumor-associated mortality in different types of cancer. This is particularly true for uveal melanoma (UM), which almost exclusively metastasizes to the liver. Hepatic stellate cells (HSCs) are the precursors of tumor-associated fibroblasts and support the growth of metastases. However, the underlying mechanisms are widely unknown. Fibroblast growth factor (FGF) signaling is dysregulated in many types of cancer. The aim of this study was to analyze the pro-tumorigenic effects of HSCs on UM cells and the role of FGFs in this crosstalk. Conditioned medium (CM) from activated human HSCs significantly induced proliferation together with enhanced ERK and JNK activation in UM cells. An in silico database analysis revealed that there are almost no mutations of FGF receptors (FGFR) in UM. However, a high FGFR expression was found to be associated with poor survival for UM patients. In vitro, the pro-tumorigenic effects of HSC-CM on UM cells were abrogated by a pharmacological inhibitor (BGJ398) of FGFR1/2/3. The expression analysis revealed that the majority of paracrine FGFs are expressed by HSCs, but not by UM cells, including FGF9. Furthermore, the immunofluorescence analysis indicated HSCs as a cellular source of FGF9 in hepatic metastases of UM patients. Treatment with recombinant FGF9 significantly enhanced the proliferation of UM cells, and this effect was efficiently blocked by the FGFR1/2/3 inhibitor BGJ398. Our study indicates that FGF9 released by HSCs promotes the tumorigenicity of UM cells, and thus suggests FGF9 as a promising therapeutic target in hepatic metastasis.

## 1. Introduction

Melanoma is a highly aggressive malignancy derived from melanocytes, pigment-producing cells of the skin. In about 5% of all melanoma cases, the tumor arises in the pigmented layer of the ocular globe encompassing the choroid, ciliary body, and iris, and is then referred to as uveal melanoma [1]. Uveal melanoma represents the most common intraocular malignancy, with an overall incidence of 5.1 cases per million per year [2].

Hepatic metastases are critical determinants of the morbidity and mortality of melanoma patients [3,4]. Overall, the liver is the second most common organ involved in cancer metastasis, after the lymph nodes. For uveal melanoma, the rate of hepatic metastasis is particularly high; in this type of cancer, 90% of metastases are hepatic [5].

It remains elusive why the liver as a metastatic niche is highly preferable for UM. Generally, the mechanisms causing the high occurrence of metastasis in the liver are largely unknown. The liver may be prone to arrest circulating cancer cells because of the slow and convoluted blood flow through the sinusoidal capillaries. Besides these hemodynamic features, the liver’s specific microenvironment may play a significant role in making it one of the most targeted organs through metastasizing cancer cells.

One of the major contributors to this prometastatic microenvironment are hepatic stellate cells (HSCs) [6]. HSCs are specialized pericytes that are quiescent, non-dividing, and vitamin A storing cells in the healthy liver [7]. Following liver injury, HSCs get activated and transdifferentiate into myofibroblasts. Upon activation, they acquire dramatic phenotypic changes, especially increased mobility and proliferation, as well as an increased production of growth factors and extracellular matrix components. Thus, HSC activation is considered as the key event of hepatic fibrosis in chronic liver disease [7].

Moreover, HSC activation also occurs in response to the colonisation and growth of metastasizing cancer cells, and there are accumulating in vitro and in vivo data indicating that HSCs promote the growth and survival of tumor cells in the liver [6]. These effects have been shown for different types of cancer that metastasized to the liver, e.g., colon carcinoma [8], pancreatic cancer [9], or breast cancer [10]. We and others have shown that HSCs also act in a pro-tumorigenic manner on melanoma cells [11,12]. However, the interaction with uveal melanoma cells and the molecular mechanisms and factors involved in the pro-tumorigenic HSC effects are widely unknown.

Fibroblast growth factors (FGFs) are important modulators of cellular proliferation, migration, and differentiation. The FGF-family is a group of 22 members, which can be divided into subgroups with intracrine, endocrine, and paracrine mechanisms of action (14). The 15 paracrine FGFs are secreted into the interstitium and act on nearby target cells as local signals via diffusion. FGFs carry out their diverse functions by binding and activating receptor tyrosine kinase (RTK) family members: the four highly conserved fibroblast growth factor receptors (FGFR) 1–4 [13]. FGF signaling has been shown to be dysregulated in many diseases, including different types of cancer [14]. The FGF/FGFR system has also been shown to play a critical role in melanoma; however, so far mostly autocrine effects and effects on local growth but not metastasis have been analyzed [15,16].

The aim of this study was to assess the pro-tumorigenic effects of HSCs on uveal melanoma cells and to dissect the role of FGFs/FGFRs in this context.

## 2. Results

### 2.1. Interaction between Hepatic Stellate Cells and Uveal Melanoma Cells

**Figure 1:**
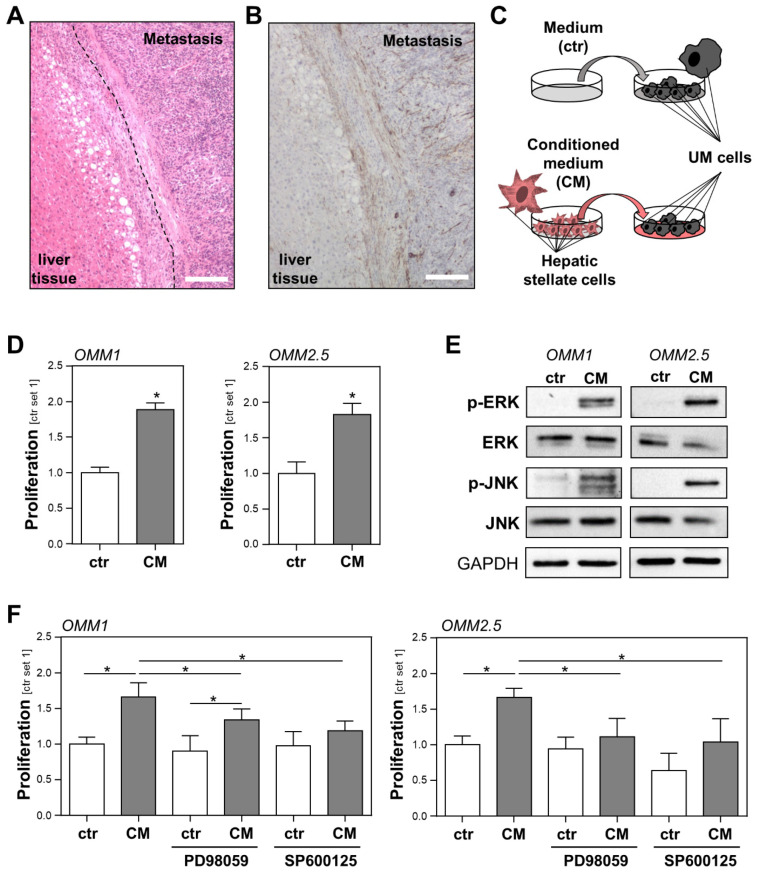
Interaction of activated hepatic stellate cells (HSCs) and uveal melanoma (UM) cells. (**A**) Hematoxylin and eosin staining of a hepatic metastasis tissue section of a patient with UM. The dashed line marks the fibrotic tissue that forms a border between the metastatic and liver tissue. Round white cavities in the liver cells represent lipid droplets and indicate that in this case, metastases developed in a slightly steatotic liver (scale bar: 200 µm). (**B**) Immunohistochemical staining of the relevant liver tissue section as shown in panel A for alpha-smooth muscle actin (α-sma), a specific marker for activated HSCs. The representative image shows the localization of HSCs in the fibrotic tissue surrounding the metastasis, as well as in its stroma (scale bar: 200 µm). (**C**) Schematic illustration of the experimental design for simulating the interaction between HSCs and UM cells in vitro. UM cells were incubated with either conditioned medium (CM) from activated HSCs or the control medium (ctr). (**D**) Proliferation of UM cell lines OMM1 (left panel) and OMM2.5 (right panel) stimulated with HSC-CM or the control medium (ctr) for 48 h. (**E**) Western blot analysis of phosphorylated ERK and phosphorylated JNK in OMM1 and OMM2.5 cells that were treated with HSC-CM or the control medium (ctr.) for 1 h. (**F**) Effects of PD98059 (inhibitor of the MEK/ERK pathway; 2 μM) and SP600125 (JNK inhibitor; 2 μM) on the HSC-CM-induced proliferation of OMM1 cells (left panel) and OMM2.5 cells (right panel). Proliferation was assessed 48 h after the stimulation of cells (*: *p* < 0.05).

Initially, we analyzed the localization of activated hepatic stellate cells (HSCs) in liver metastases from uveal melanoma (UM) patients (Figure 1A and Figure S1). Immunohistochemical staining of alpha-smooth muscle actin (α-sma), an established marker for activated HSCs [17], showed that these cells surround and also form the stroma of the metastases, indicating the close contact between HSCs and UM cells (Figure 1B and Figure S1). To simulate the interaction between activated HSCs and UM cells in vitro, we stimulated metastatic UM cell lines with conditioned medium (CM) collected from activated HSCs (HSC-CM) (Figure 1C). Serum-free media incubated in cell culture flasks without cells was used to stimulate UM cells, which were then denoted as the controls. In the functional analysis, HSC-CM significantly induced the proliferation of the UM cell lines OMM1 and OMM2.5 (Figure 1D). Activation of the mitogen-activated protein kinases (MAPK) extracellular-signal regulated kinase (ERK) and c-Jun N-terminal kinase (JNK) are implicated in the proliferation of (uveal) melanoma [18,19]. Therefore, we analyzed the effect of HSC-CM on the phosphorylation of ERK and JNK in UM cells. Stimulation with HSC-CM markedly induced ERK and JNK phosphorylation in OMM1 and OMM2.5 cells (Figure 1E). Treatment with HSC-CM also resulted in increased phosphorylation of ERK and JNK in the primary tumor cell line Mel270 (Figure S2), indicating that HSC-CM effects are not restricted to metastatic UM cell lines. To investigate whether CM-induced activation of ERK or JNK is responsible for the observed growth promoting effects of HSCs on metastatic UM cells, incubation with HSC-CM was performed with specific inhibitors of the MEK/ERK (PD98059) or the JNK pathway (SP600125). In OMM1 cells, incubation with SP600125 inhibited the HSC-CM-induced proliferation (Figure 1F). The ERK inhibitor PD98059 also reduced the HSC-CM effect in OMM1 cells compared with the OMM1 cells incubated with HSC-CM alone (Figure 1F). Nevertheless, OMM1 cells treated with the combination of PD98059 and HSC-CM still showed significantly higher proliferation than OMM1 cells treated with PD98059 alone (Figure 1F). In OMM2.5 cells, PD98059 almost completely abrogated HSC-CM-induced proliferation (Figure 1F). Treatment with SP600125 reduced both basal and HSC-CM induced proliferation in OMM2.5 cells (Figure 1F). The observation that stimulation with the JNK inhibitor SP600125 led to diminished basal proliferation of OMM2.5 cells suggests that OMM2.5 cells secrete autocrine factors that sustain proliferation via JNK signaling. Although SP600125 almost completely blocked the HSC-CM-induced proliferation of OMM2.5 cells when compared with the control cells treated with HSC-CM alone (Figure 1F), OMM2.5 treated with HSC-CM and SP600125 still showed a higher proliferation than OMM2.5 cells treated with SP600125 only. Together, these data indicate that soluble factors secreted from HSCs promote the proliferation of UM cells, and that the activation of both ERK and JNK signaling plays a role in this growth promoting effect.

### 2.2. Role of the FGF/FGFR System in the Prognosis of Uveal Melanoma (UM) Patients and in the Interaction between Hepatic Stellate Cells (HSCs) and UM Cells

Fibroblast growth factors (FGFs) are pleiotropic factors that exert autocrine and paracrine functions on tumor and stromal cells, and the FGF/FGF receptor (FGFR) system has been demonstrated to contribute to the progression of several types of cancer [20,21]. Therefore, we hypothesized that the FGF/FGFR system might play a role in the interaction between HSCs and UM cells. Notably, an analysis of the COSMIC (Catalogue of Somatic Mutations in Cancer) database (v95, released 24 November 2021) of 221 tissue samples from patients with melanomas of the uveal tract of the eye revealed no mutations in *FGFR1*, *FGFR2*, or *FGFR3*, and only one mutation in *FGFR4* (c.514T>G), respectively. In contrast, genetic alterations, such as amplifications, mutations, and translocations in *FGFR* have been found to play a role in the initiation and progression of several types of cancer, most commonly non-small cell lung cancer, breast cancer, glioblastoma, prostate cancer, and gastrointestinal cancer [22,23,24]. 

**Figure 2:**
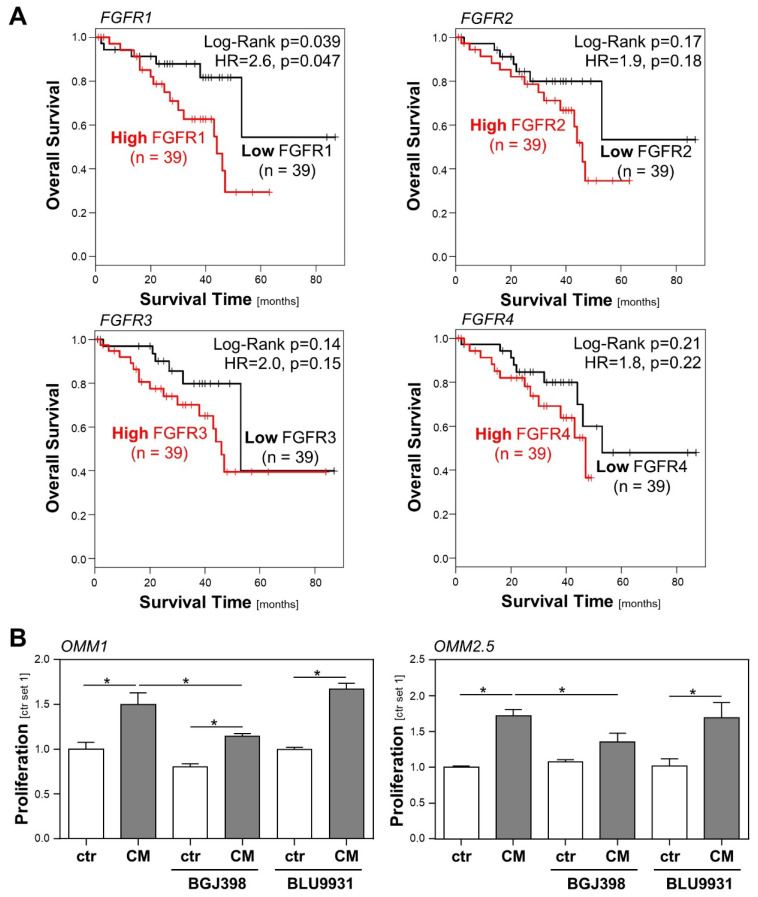
Role of the FGF/FGFR system in the prognosis of uveal melanoma (UM) patients, and in the interaction between hepatic stellate cells (HSCs) and UM cells. (**A**) Correlation between high/low *FGFR1-4* expression and overall survival of UM patients applying a TCGA dataset of 78 UM patients and GEPIA database. (**B**) Effects of BGJ398 (FGFR1-3 inhibitor; 200 nM) and BLU9931 (FGFR4 inhibitor; 100 nM) on HSC-CM-induced proliferation of OMM1 cells (left panel) and OMM2.5 cells (right panel). Proliferation was assessed 48 h after the stimulation of cells (*: *p* < 0.05).

However, an analysis of a TCGA dataset of 78 UM patients applying the GEPIA platform [25] revealed that a high *FGFR* expression is associated with a poor survival of UM patients (Figure 2A). Although only the correlation of a high *FGFR1* expression with survival reached the level of significance, these data indicate that the *FGFR* expression may impact UM progression, and that rather than activating mutations, FGFR ligands may be involved.

In order to investigate whether FGFR activation might be implicated in the CM-induced proliferation of UM cells, we compared the effects of the selective FGFR4 inhibitor BLU9931 [26] and the FGFR1/2/3 inhibitor BGJ398 (infigratinib) [27] regarding the growth promoting effect of HSC-CM on UM cells. In OMM1 cells, HSC-CM-induced proliferation was significantly reduced by BGJ398, whereas BLU9931 exhibited no significant effect (Figure 2B). Still, BGJ398 also had a slight but non-significant effect on the basal proliferation of OMM1 cells, and in the presence of BGJ398, HSC-CM-induced proliferation was not completely inhibited (Figure 2B). This indicates that there might also be a low autocrine secretion of FGFR1/2/3 ligands by OMM1 cells, and that HSC-CM also mediates its effects by other growth factors besides FGFs. Similarly, HSC-CM-induced proliferation was significantly, but not completely, inhibited by BGJ398 in OMM2.5 cells, whereas BLU9931 exhibited no significant effect (Figure 2B). These data indicate that FGFR1/2/3 ligands secreted by HSCs significantly contribute to the CM-induced tumorigenicity of UM cells.

### 2.3. Identification of FGF9 as a Factor Secreted by Hepatic Stellate Cells That Promotes the Tumorigenicity of Uveal Melanoma Cells

**Figure 3:**
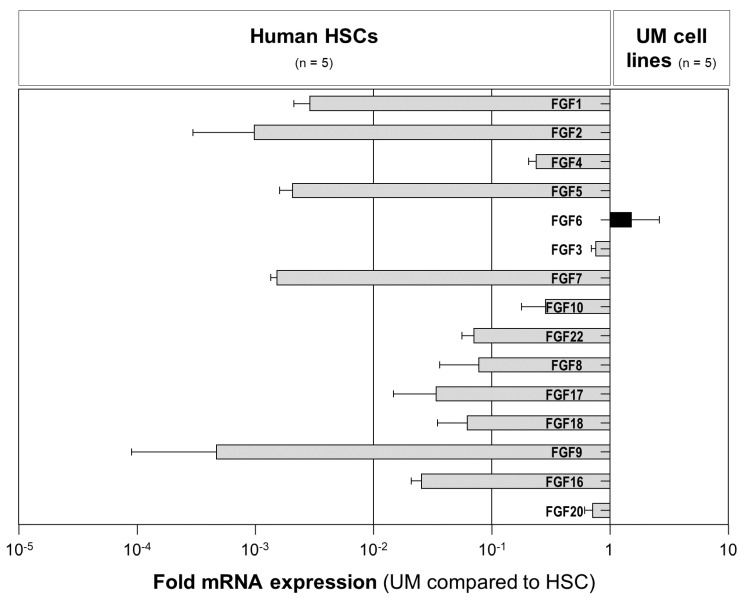
The expression of paracrine FGFs in human hepatic stellate cells (HSCs) and uveal melanoma (UM) cells. Fold mRNA expression levels of paracrine FGFs (subfamilies 1 (FGF1 and FGF2), 4 (FGF4, FGF5, and FGF6), 7 (FGF3, FGF7, FGF10, and FGF22), 8 (FGF8, FGF17, and FGF18), and 9 (FGF9, FGF16, and FGF20)) in five human UM cell lines (Mel270, OMM1, OMM2.3, OMM2.5, and OMM2.6) compared with the expression in the primary human HSCs from five different donors. Data are shown as the mean ± standard error of the mean (SEM).

To screen for potential FGFs that are secreted by activated HSCs and that might promote the tumorigenicity of UM cells, we analyzed the mRNA expression of paracrine FGFs subfamilies 1 (FGF1 and FGF2), 4 (FGF4, FGF5, and FGF6), 7 (FGF3, FGF7, FGF10, and FGF22), 8 (FGF8, FGF17, and FGF18), and 9 (FGF9, FGF16, and FGF20) in activated human HSCs from five different human donors and in five human UM cell lines (Mel270, OMM1, OMM2.3, OMM2.5, and OMM2.6) using qRT-PCR analysis. The expression levels of all paracrine FGFs, except FGF6, were significantly higher in activated human HSCs compared with UM cell lines (Figure 3).

**Figure 4:**
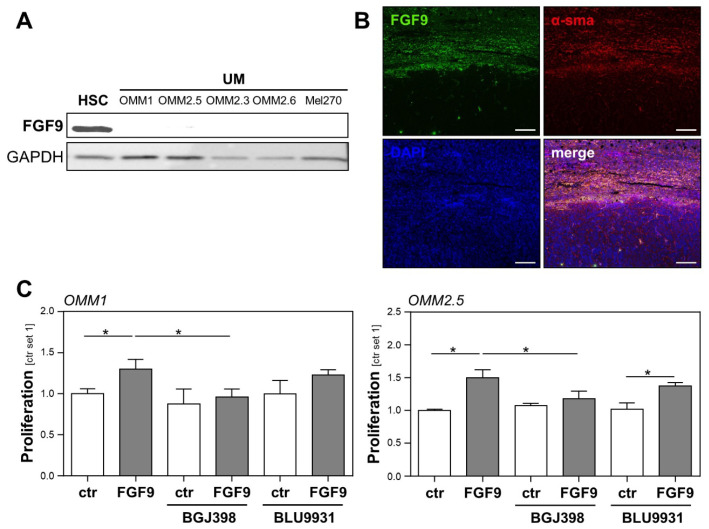
Expression of FGF9 in hepatic metastasis from uveal melanoma (UM) and the effect of FGF9 on the tumorigenicity of UM cells. (**A**) FGF9 protein expression (analyzed by Western blot) in primary human hepatic stellate cells (HSC) and human UM cell lines (OMM1, OMM2.5, OMM2.3, OMM2.6, and Mel270). (**B**) Immunofluorescence staining for FGF9 (green) and alpha-smooth muscle actin (α-sma, red) in human liver metastasis from UM. Nuclei were counterstained using DAPI (blue; scale bar: 200 µm). (**C**) Effects of BGJ398 (FGFR1/2/3 inhibitor, 100 nM) and BLU9931 (FGFR4 inhibitor, 100 nM) on the FGF9-induced proliferation of OMM1 (left panel) and OMM2.5 (right panel) cells. Proliferation was assessed 48 h after the stimulation of cells (*: *p* < 0.05).

We further focused our analyses on FGF9, which showed the highest expression levels in HSCs (Figure 3). Western blot analysis confirmed a higher FGF9 protein abundance in HSCs than in UM cell lines (Figure 4A). In line with this, the immunofluorescence analysis revealed colocalization of α-sma and FGF9 in the liver metastases from UM patients, further supporting the view that HSCs are the cellular source of FGF9 in hepatic metastasis from UM (Figure 4B).

Stimulation with recombinant FGF9 induced the phosphorylation of ERK in OMM1 and OMM2.5 cells (Figure S3). Surprisingly, no effect on JNK phosphorylation in OMM1 cells was observed following treatment with recombinant FGF9, although stimulation with recombinant FGF9 slightly induced the phosphorylation of JNK in OMM2.5 cells (Figure S3).

To further evaluate the effect of FGF9 on the proliferation of UM cell lines and to test whether FGF9 effects could be blocked by FGFR inhibition, UM cell lines OMM1 and OMM2.5 were stimulated with recombinant FGF9, in combination with the specific FGFR1/2/3 inhibitor BGJ398 and the specific FGFR4 inhibitor BLU9931. FGF9 induced the proliferation of OMM1 and OMM2.5 cells, and this effect was completely blocked by the FGFR1/2/3 inhibitor BGJ398, whereas the FGFR4 inhibitor BLU99331 had no significant effect (Figure 4C). Together, these data indicate that FGF9 might be a substantial factor through which activated HSCs induce the proliferation of UM cells, and that blocking the FGF9-induced FGFR1/2/3 activation might represent a potential therapeutic option for UM patients presenting with hepatic metastasis.

## 3. Discussion

Uveal melanoma (UM) is characterized by its uniquely high propensity to spread to the liver [5]. The molecular mechanisms promoting this phenomenon are elusive, as generally, it is widely unknown why the liver is so attractive for metastatic growth. Hepatic stellate cells (HSCs) are the precursors of tumor-associated fibroblasts not only in hepatocellular carcinoma [28], but also in the liver metastasis of various tumor entities [9], and it is known that activated HSCs promote the growth of metastases [6]. However, the mechanisms through which HSCs exert metastasis-promoting functions are poorly understood, especially in UM.

Here, we found that activated HSCs do not only surround the metastases of UM, but also form the stroma of the metastases. This was very similar, as described in previous studies [29,30], and indicated the close contact and potential interaction between HSCs and UM cells. Indeed, we found that primary human HSCs secrete soluble factors that induce the proliferation of UM cell lines in vitro. In contrast, in a study by Babchia et al., no growth promoting effects of HSCs on UM cell lines were observed [31]. This divergence might be explained by the fact that Babchia et al. used the immortalized cell line LX-2 [31]. Although primary HSCs and LX-2 cells are substantially similar, there are distinct differences in the gene expression profile [32]. Thus, the use of primary HSCs in our study might more closely resemble the clinical situation.

As it is the potential molecular mechanism through which HSCs mediate growth-promoting effects on UM cells, we identified an increased activity of extracellular-signal regulated kinase (ERK), similarly to that observed by Cheng et al. [11]. Generally, the MEK/ERK axis is known as a core module in controlling the proliferation and survival of UM cells [19]. However, data in the literature are controversial. Ambrosini et al. demonstrated that MEK inhibition with selumitinib led to reduced cell viability of GNAQ/GNA11-mutant UM cell lines [33]. Interestingly, GNAQ/GNA11-mutant UM cell lines were less sensitive to MEK inhibition than BRAF-mutant UM cells [33]. Importantly, their results showed that MEK inhibition resulted in upregulation of c-Jun in GNAQ/GNA11-mutant UM cell lines, possibly representing an alternative route to cell proliferation—an observation that might explain the lower sensitivity of GNAQ/GNA11-mutant UM cell lines towards MEK inhibition than BRAF-mutant UM cells [33]. In a publication by Faião-Flores et al., a panel of GNAQ/GNA11-mutant uveal melanoma cell lines (including OMM1 cells) was characterized with respect to the MEK inhibitor response (i.e. trametinib) [34]. In contrast with the study by Ambrosini et al. [33], they found that MEK inhibition only modestly inhibited the proliferation of UM cell lines [34]. In line with this, stimulation with trametinib also resulted in only a little induction of apoptosis [34]. In our study, the MEK/ERK inhibitor PD98059 merely and non-significantly inhibited the proliferation of UM cell lines OMM1 and OMM2.5, in agreement with the results of Faião-Flores et al. [34].

Moreover, we found that HSCs secrete factors that induce the activation of c-Jun N-terminal kinase (JNK) in UM cells. Importantly, we and others have shown that JNK activation plays a critical role in melanoma development and progression [18,35]. However, little information is available regarding the effects of JNK inhibition on the proliferation of UM cells. In a study by Zhu et al., the JNK inhibitor SP600125 had no impact on the proliferation of the human choroidal melanoma cell line MEL15-1 after 48h of treatment [36]. In line with this, the knockdown of c-jun had only a modest effect on the basal proliferation of UM cells, as demonstrated in a study by Khalili et al. [37]. In line with this, in our study, the JNK inhibitor SP600125 had no effect on OMM1 cell growth at baseline and in OMM2.5 cells, and the JNK inhibitor had only a little effect on basal cell proliferation (not significant). Importantly, in our study, serum-free medium was used and pathway inhibitors might have more pronounced effects in the medium containing serum.

In the search for relevant factors mediating HSC-CM induced proliferation of UM cells, we focused on the fibroblast growth factor (FGF)/FGF receptor (FGFR) system, as we previously demonstrated that activated HSCs are the major cellular source of most paracrine FGFs in hepatocellular cancer [38], and the FGF/FGFR axis is known to play a pivotal role in various tumor entities [20,21]. In melanoma, FGF signaling has been mainly studied in the interaction between tumor cells with fibroblasts and keratinocytes, autocrine effects, and in local growth and angiogenesis [16]. Here, we found that different than in many other cancer types, FGFRs are not mutated in UM. However, we found that a high FGFR expression was associated with a lower survival rate for UM patients. Although only the correlation between a high FGFR1 expression and survival reached the level of statistical significance, the data indicate that FGFRs affect the prognosis of UM patients. As the latter is critically determined by hepatic metastasis, we further speculated that FGFR ligands in the hepatic environment contribute to this correlation. In line with this hypothesis, we demonstrated that FGFR1/2/3 inhibition reduced the growth promoting effect of conditioned media from HSCs on UM cells, indicating that this HSC effect is mediated by soluble FGFR1/2/3 ligands. In a further analysis, we focused on FGF9, which was found to be the most highly expressed by primary human HSCs in vitro. Coimmunofluorescence analysis of FGF9 and the specific HSC marker α-sma further supported the view that HSCs are the cellular source of FGF9 in hepatic UM metastases. Furthermore, we found that FGF9 promoted the proliferation of UM cell lines in vitro. Together, these data point towards the role of FGF9 as a HSC-derived factor that promotes the tumorigenicity of UM cells and herewith hepatic metastasis. Thus, the inhibition of FGF9 might be a potential therapeutic approach for the treatment of hepatic metastasis from UM. Currently, there are significant improvements in the development of specific strategies to block defined FGFs, such as FGF ligand traps or antibodies [20,21]. Furthermore, BGJ398 (infigratinib) has already been tested for the treatment of patients with advanced solid tumors in clinical studies [39]. Thus, also targeting the FGFR1/2/3 axis might represent a potential therapeutic strategy for UM patients with hepatic metastasis.

The HSC effects on FGFR activation may potentially also have an impact on the therapy resistance of hepatic metastasis, as growing evidence suggests that FGF/FGFR signaling confers resistance to oncotherapy in various types of cancer (as reviewed in [40]). A previous study has shown that FGF2 produced by HSCs mediates the resistance of metastatic UM to bromodomain and extraterminal (BET) protein inhibitors via FGFR activation [41]. Furthermore, we demonstrated that FGF9 secreted by activated HSCs reduces the sensitivity of HCC cells against the multi-tyrosine kinase inhibitor sorafenib [38]. Thus, it might be speculated that HSC-derived FGF9 is also implicated in the therapy resistance of metastatic UM. Accordingly, FGF traps or anti-FGF antibodies [20] could be exploited to specifically target FGF9 alone or in combination with other drugs, such as BET or FGFR inhibitors. This aspect should be addressed in further studies. One limitation of the present study is that the functional effects of HSCs and FGF9, respectively, on UM cells have only been studied in vitro. Recently, Piquet et al. described an elegant xenograft model in which they studied the synergistic interactions between HSCs and UM cells on metastatic growth [29]. It would be very interesting to further validate the role of FGF9 in this model and whether it contributes to the induction of the pre-metastatic niche in vivo. In this context, it would be tempting to speculate whether the modulation of FGF9 signaling by FGFR inhibitors, FGF traps, or anti-FGF antibodies could be exploited as a therapeutic strategy.

In the present study, FGFs were identified as substantial, but not the only factors through which HSCs promote the tumorigenicity of UM cells. Previous studies have illustrated that other growth factors, such as hepatocyte growth factor (HGF), released by HSCs protects metastatic UM cells from the growth inhibitory effects of MEK inhibition [11,42]. Furthermore, several lines of evidence indicate that insulin-like growth factor I (IGF-1) is also implicated in UM spreading to the liver [43,44]. These studies further underscore the role of HSCs in the hepatic metastasis of UM.

A recent and elegant review by Loda et al. extensively summarized the relevant roles of FGF/FGFR signaling in ocular tumors, including UM [45]. As reviewed by Loda et al., the inhibition of FGF/FGFR signaling might also play an integral part in the tumor/stroma interaction as an anti-angiogenic strategy, given the importance of FGFs in neo-angiogenesis [45]. It is tempting to speculate whether FGF9 or other factors secreted by HSCs also have an impact on the angiogenesis of hepatic UM metastases. This aspect should be addressed in future studies. In line with the results of the present study, Loda et al. proposed FGF/FGFR-targeted therapies as an integrate approach that is suitable to treat UM with regard to both stromal and parenchymal compartments [45].

In conclusion, our findings indicate that HSC-derived factors enhance the tumorigenicity of UM cells, and that FGFs play a major role in this pro-tumorigenic setting. In this context, FGF9 has been identified as a new potential therapeutic target in the hepatic metastasis of UM. These data form the basis for future studies elucidating the role of FGF9 in liver metastasis, and may open venues for the accurate and tightly regulated modulation of FGF-signaling in patients with hepatic metastasis from UM.

## 4. Materials and Methods

### 4.1. Cells and Cell Culture

Human UM cell lines Mel270 [46], OMM1 [47], OMM2.3 [48], OMM2.5 [48], and OMM2.6 [46] were cultured as described [47,48,49]. Mel270 (primary tumor), OMM2.3, OMM2.5, and OMM2.6 (metastases; liver) were derived from patient 270 and the history of these cell lines was extensively described in a publication by Jager et al. [50]. OMM1 cells were derived from a subcutaneous metastatic lesion of another patient [47]. For our in vitro model, UM cell lines of a metastatic origin derived from two different patients were chosen: OMM2.5 cells were derived from the liver metastasis of UM [48], whereas OMM1 cells were derived from a subcutaneous metastatic lesion [47].

Human liver cell suspensions were prepared by a two-step collagenase perfusion procedure with modifications as described [51,52], and were provided by the non-profit state-controlled HTCR (Human Tissue and Cell Research) foundation [53]. Tissue samples for cell isolation were obtained from patients undergoing partial hepatectomy, e.g., for metastatic liver tumors. Only those liver tissues judged as “normal” by local pathologists were used for cell preparation.

All of the experimental procedures were performed according to the guidelines of the non-profit state-controlled HTCR (Human Tissue and Cell Research) foundation [53]. The study was performed according to the principles of the Declaration of Helsinki and was approved by the HTCR Review Board. Informed consent was obtained from all subjects involved in the study.

The isolation and cultivation of the primary human HSC was performed as described [54]. HSCs were cultured on uncoated tissue culture dishes to induce spontaneous activation. Activation of HSC was checked by assessing the alpha-smooth muscle actin expression via Western blotting (Figure S4).

For the collection of the conditioned medium (CM) from activated HSCs, activated HSCs were seeded into T75 cell culture flasks (1 × 10^6^ cells). The next day, HSCs were washed with serum-free DMEM and were then cultivated in serum-free DMEM (10 mL/T75 flask) for 24 h. Serum-free DMEM incubated in T75 cell culture flasks without cells served as the control. The conditioned medium and control medium were collected, centrifuged for 10 min to remove the cell debris, and stored at –20 °C until use. The conditioned medium or control medium were added, undiluted to the UM cells for the experiments (1 mL/well in 6-well plates or 100 µL/well in 96-well plates).

For the individual experiments, the cells were treated with recombinant human FGF9 (R&D Systems, Minneapolis, MN, USA), PD98059 (inhibitor of the MEK/ERK pathway; Calbiochem, La Jolla, CA, USA), SP600125 (JNK inhibitor; Calbiochem, La Jolla, CA, USA), the selective FGFR1-3 inhibitor BGJ398 (Selleckchem, Munich, Germany), or the selective FGFR4 inhibitor BLU9931 (Cayman Chemicals, Ann Arbor, MI, USA).

### 4.2. Analysis of Cell Proliferation

Proliferation of cells was determined using a colorimetric XTT assay (Roche Diagnostics, Mannheim, Germany) according to the manufacturer’s instructions [55].

### 4.3. Protein Analysis

Protein extraction and Western blotting were performed as described [55] using the following primary antibodies: rabbit anti-phospho-ERK (#9101, 1:1000; Cell Signaling Technology, Danvers, MA, USA), rabbit anti-p44/42 MAPK (ERK) (#9102, 1:1000; Cell Signaling Technology, Danvers, MA, USA), rabbit anti-phospho-JNK (#9251, 1:1000; Cell Signaling Technology, Danvers, MA, USA), mouse anti-JNK1 (#3708, 1:1000; Cell Signaling Technology, Danvers, MA, USA), rabbit anti-FGF9 (PA5-103855, 1:1000; Invitrogen, Thermo Fisher Scientific, Waltham, MA, USA), rabbit anti-alpha smooth muscle actin (ab32575, 1:1000; Abcam, Cambridge, UK), and rabbit anti-GAPDH (#2118, 1:1000; Cell Signaling Technology, Danvers, MA, USA). Mouse anti-rabbit (sc-2357, 1:10,000, Santa Cruz Biotechnology, Dallas, TX, USA) and horse anti-mouse (#7076, 1:3000; Cell Signaling Technology, Danvers, MA, USA) were used as the secondary antibodies.

### 4.4. Hematoxylin and Eosin Staining and Immunohistochemical Staining

For hematoxylin and eosin staining and for immunohistochemical staining, standard sections of formalin-fixed and paraffin-embedded patient-derived UM tissues (*n* = 6) were used. Immunohistochemical staining for alpha-smooth muscle actin (α-sma) was performed applying anti-α-sma antibody (ab32575, 1:300; Abcam, Cambridge, MA, USA) and standard protocols as described [56].

### 4.5. Immunofluorescence Staining

Sections of formalin-fixed and paraffin-embedded patient-derived UM tissues were also used for immunofluorescence staining, applying anti-FGF9 antibody (AF-273-NA, 1:25; R&D Systems, Minneapolis, MN, USA) and anti-α-sma antibody (ab32575, 1:500; Abcam) and standard protocols as described [29]. The following secondary antibodies were used: Alexa Fluor 488-conjugated donkey anti-goat IgG (A11055, 1:1000; Invitrogen, Thermo Fisher Scientific, Waltham, MA, USA) and Cy3-conjugated donkey anti-rabbit IgG (711-165-152, 1:1000; Jackson ImmunoResearch Laboratories, Inc., West Grove, PA, USA). Nuclei were counterstained using DAPI.

### 4.6. Analysis of mRNA Expression

Isolation of mRNA, reverse transcription, and quantitative real-time polymerase chain reaction (qRT-PCR) were performed as described [38]. The specific sets of primers that were used are summarized in Table 1. For the detection of human FGF1, FGF3, FGF4, FGF16, FGF17, and FGF22 genes, QuantiTect Primer assays (Qiagen, Hilden, Germany) were used. Amplification of cDNA derived from GAPDH was used for normalization of the data.

### 4.7. In Silico Analysis

The Gene Expression Profiling Interactive Analysis (GEPIA) platform [25] was used to evaluate the prognostic value of FGFR expression with respect to the overall survival of uveal melanoma patients in a TCGA dataset of 78 UM patients. UM patients were divided into high and low FGFR expression groups using a median cutoff.

### 4.8. Statistical Analysis

Statistical analysis was carried out using GraphPad Prism Software version 6.01 (GraphPad Software, San Diego, CA, USA). Data are shown as the mean ± standard deviation (SD), unless stated otherwise. Data sets were compared through an analysis of the unpaired Student’s t-test or one-way ANOVA for multiple comparison tests when appropriate. A *p*-value < 0.05 was considered statistically significant.

## Figures and Tables

**Table 1:** Primer sequences for qRT-PCR.

Gene	Forward (5′-3′)	Reverse (5′-3′)
FGF2	GCGACCCTCACATCAAGCTACA	CTGCCCAGTTCGTTTCAGTGC
FGF5	CAGCAGTAGCGCTATGTCTTCCT	TACAATCCCCTGAGACACAGCA
FGF6	AGTGCCCTCTTCGTTGCCAT	CCCGCTTTACCCGTCCGTAT
FGF7	GGCAATCAAAGGGGTGGA	CCTCCGTTGTGTGTCCATTTA
FGF8	AGGGTGTCTCCCAACAGGTAAC	GGTGTCCGTCTCCACGATGA
FGF9	ATTTCGGTGTGCAGGATGCG	CTGACCAGGCCCACTGCTAT
FGF10	GAGTTGTTGCCGTCAAAGCCA	TTGCCTCCCATTATGCTGCCA
FGF18	ATGGGGACAAGTATGCCCAGC	TGGTGAAGCCCACGTACCAG
FGF20	CTATTGCCGCACCGGCTTC	CCACAAAATACCTGCGGCCAG
GAPDH	GGCTCTCCAGAACATCATCCCTGC	GGGTGTCGCTGTTGAAGTCAGAGG

## Data Availability

The data that support the findings of this study are available from the corresponding author upon reasonable request.

## Supplementary Materials

The following supporting information can be downloaded at: https://www.mdpi.com/article/10.3390/ijms231911524/s1.
