# Supplementary material for: Role of Fibroblast Growth Factors in the Crosstalk of Hepatic Stellate Cells and Uveal Melanoma Cells in the Liver Metastatic Niche
Source: Int J Mol Sci. 2022 Sep 29;23(19):11524. doi: 10.3390/ijms231911524 (PMC9569506; doi:10.3390/ijms231911524)

Supplementary Figures

Role of fibroblast growth factors in the crosstalk of hepatic stellate cells and uveal melanoma cells in the liver metastatic niche

Tatjana Seitz, Nora John, Judith Sommer, Peter Dietrich, Wolfgang E. Thasler, Arndt Hartmann, Katja Evert, Sven A. Lang, Anja Bosserhoff and Claus Hellerbrand

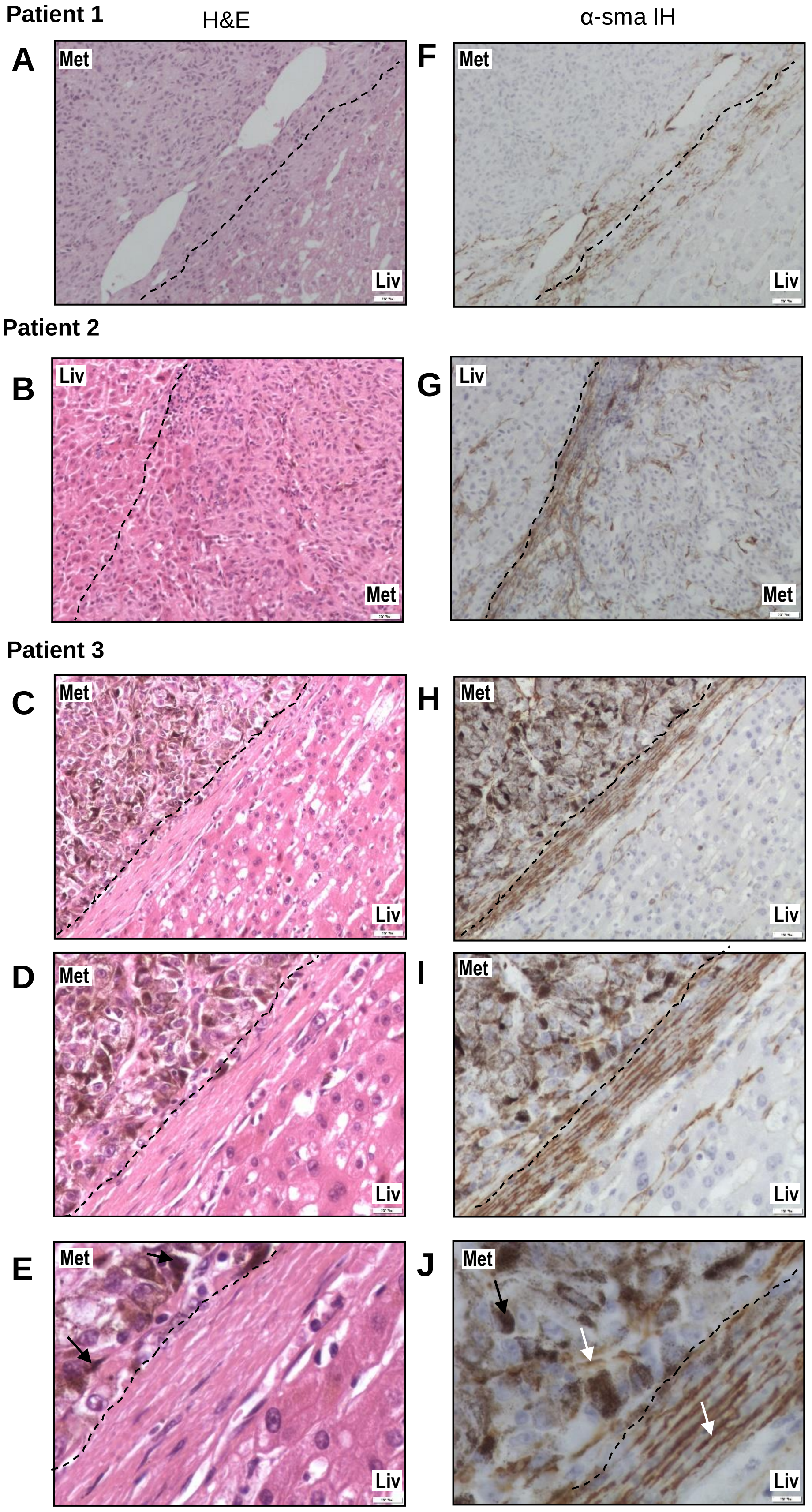

Supplementary Figure S1. Interaction of activated hepatic stellate cells (HSCs) and uveal melanoma (UM) cells.

(A-E) Hematoxylin and eosin (H&E) and (F-J) alpha-smooth muscle actin (α-sma) immunohostochemical (IH) stainings of tissue sections of hepatic metastases of three patients with UM. The dashed lines mark the fibrotic tissue that forms a border between the metastatic and the liver tissue. Typical trabecular structure and lining of the hepatocytes along the liver sinusoids are characteristic for normal non-tumorous liver tissue (Liv). In contrast, disorganized cells with irregular nuclei are typical for tumor cells forming the large part of the hepatic metastasis (Met). Spindle-shaped cells in fibrotic septa between Liv and Met are typical for activated hepatic stellate cells (HSCs). Tumor cells in the metastatic tissue of patient 3 can be additionally recognized by dark brown color in H&E staining (C-E), which is typical for melanin granules in melanoma cells. In contrast, tumor cells in metastases of patients 1 (A) and 2 (B) show no brown staining indicative for a loss of melanin production in these melanoma cells, which is frequently observed during malignant transformation. Despite the inherent brown staining of tumor cells (examples depicted by black arrow, panels E and J), α-sma positive HSCs can be distinguished due to brighter brown coloring as well as form and staining pattern (examples depicted by white arrows in panel J), which is particularly evident at higher magnifications (magnifications: A-C and F-H: 10x; D,I: 20x; E,J 40x).

Supplementary Figure S2: Western blot analysis of phosphorylated ERK and phosphorylated JNK in Mel270 cells that were treated with HSC-CM or control medium (ctr.) for 1h.

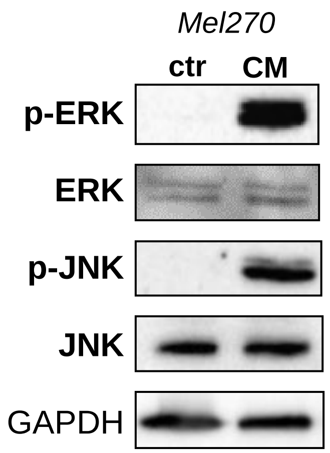

Supplementary Figure S3: Western blot analysis of phosphorylated ERK and phosphorylated JNK in OMM1 and OMM2.5 cells that were treated with recombinant FGF9 or control medium (ctr.) for 1h.

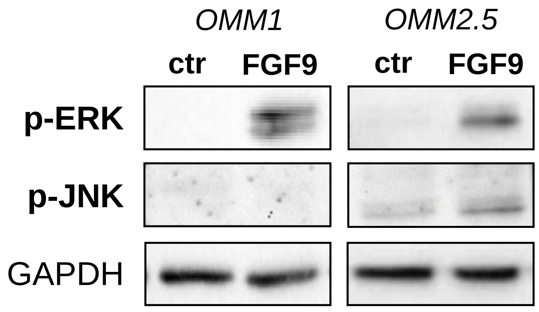

Supplementary Figure S4. Western blot analysis of alpha-smooth muscle actin (α-sma) during the course of in vitro activation of primary hepatic stellate cells.

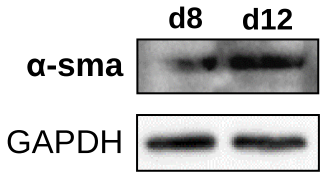

Supplement: Supplementary file 1 [file ijms-23-11524-s001.zip › ijms-1912228-supplementary.pdf]
